# Toxin and Immunotoxin Based Therapeutic Approaches

**DOI:** 10.3390/toxins14010063

**Published:** 2022-01-17

**Authors:** Massimo Bortolotti, Letizia Polito, Andrea Bolognesi

**Affiliations:** Department of Experimental, Diagnostic and Specialty Medicine—DIMES, Alma Mater Studiorum—University of Bologna, Via S. Giacomo 14, 40126 Bologna, Italy

The concept of “magic bullets”, i.e., drugs able to selectively act on target cells, formulated by Paul Ehrlich more than one century ago, gave rise to the idea of immunotargeting, one of the most studied approaches being based on antibodies carrying toxic moieties [1]. Bacterial or plant toxins can be joined to specific carriers through chemical linking or genetic engineering, antibodies being the most used carriers and the generated hybrid molecules having been named immunotoxins. These conjugates are functionally designed to eliminate pathological cells, finding applications in several fields such as cancer, immunological diseases or pain control.

Among plant toxins, the most used are ribosome-inactivating proteins (RIPs), a family of enzymes widely spread in the plant kingdom [2]. RIPs possess polynucleotide:adenosine glycosylase activity with the ability to remove adenines from several polynucleotide substrates, causing cell death. Adenine removal from *r*RNA, the first RIP activity to be described, damages ribosomes in an irreversible manner, causing the inhibition of protein synthesis; thus, explaining the origin of these proteins’ name. RIPs are mainly classified as type 1, consisting of a single-chain protein with enzymatic activity, and type 2, consisting of an enzymatic A chain linked by a disulfide bond to a lectin B chain that is able to bind sugar-containing receptors on the cell membrane. The presence of the B chain in type 2 RIPs allows for a more rapid and efficient internalization into the cell than type 1 RIPs. For this reason, type 2 RIPs are highly cytotoxic [2]. Ricin is the most widespread and well-known type 2 RIP and also the most used in the construction of immunotoxins [3]. As RIPs have different intracellular substrates and are able to elicit more than one cell death pathway, they are drugs potentially suitable for a targeted cancer treatment. Furthermore, no drug resistance against toxins has been reported so far.

Among bacterial toxins, the most used are pseudomonas exotoxin A and the diphtheria toxin, which inhibit translation through the NAD-dependent ADP-ribosylation of the elongation factor-2, causing cell death [4]. Several immunotoxins have been developed using bacterial toxins and a variety of carriers specific for different targets. Up to date, three of these conjugates have been approved by the U.S. Food and Drug Administration for hematological cancer therapy [5].

The collection of seven scientific articles composing this Special Issue highlights the progress in the knowledge of toxins and immunotoxins; thus, underlying their potential in anticancer therapy.

In this Special Issue, a review article is included concerning the application of a new cell-based IT screening system offering several advantages in the formulation of new immunotoxins by enabling the straightforward and rapid selection of novel functional antibodies [6].

A fundamental requirement for the therapeutic application of toxins and their conjugates is the knowledge of their biochemical and structural properties, as well as of their binding, uptake, intracellular routing and substrate specificities. In this Special Issue, the complete amino acid sequence and 3D structure prediction of two potent type 2 Adenia RIPs, namely, stenodactylin [7] and kirkiin [8], are determined. RIPs purified from the Adenia genus are known to be among the most lethal plant toxins [9]. The authors observed high structural and amino acid sequence homologies with other type 2 RIPs and particularly with those identified in plants belonging to the Adenia genus. The stenodactylin B chain showed a high degree of identity with B chains of other type 2 RIPs, supporting the hypothesis that the B chain is a product of a gene duplication event. A hemagglutination analysis revealed that both kirkiin and stenodactylin have similar affinities for D-galactose and lactose, although the affinity of kirkiin for these sugars was lower with respect to ricin. In both Adenia toxins, the replacement of histidine instead of ricin tyrosine in the sugar binding site of B chains was detected, possibly justifying the reduction in the sugar-binding affinity, although not seeming to affect cytotoxicity. Moreover, the cytotoxicity of quinoin, a recently purified type 1 RIP from quinoa seeds, was evaluated using human glioblastoma cell lines, and was seen to strongly reduce glioblastoma cell growth at concentrations in the nM range. Interestingly, an additive effect was found in primary cells treated with quinoin in combination with the chemotherapeutic temozolomide [10].

The Special Issue also focuses on the possibility to obtain selective and potent toxin-based conjugates able to be used for pharmacological purposes for different targets, with three interesting articles having been published in this regard. A fusion protein between the ricin A chain and pokeweed antiviral protein (RTAM-PAP1) was studied in silico for docking against various key proteins of SARS-CoV-2. The experiments revealed novel binding mechanisms of RTAM-PAP1 with a high affinity to numerous SARS-CoV-2 key proteins. RTAM-PAP1 was further characterized in a preliminary toxicity study in mice, and was found to be a potential therapeutic candidate [11]. The immunotoxin DT389-YP7 was obtained by fusing a truncated diphtheria toxin without a binding domain with a humanized YP7 scFv specific for a highly expressed Glypican-3 antigen on the surface of hepatocarcinoma cells, resulting specifically cytotoxic on the target HepG2 cell line. Cellular morphological changes, cell cycle arrest at the G2 phase, augment in radical oxygen species production, induction of apoptosis, and inhibition of cell movement were observed after the immunotoxin treatment [12]. The two recombinant anti epidermal growth factor receptor (EGFR) conjugates, EGF-PE40 and EGF-PE24mut, were constructed by fusing the epidermal growth factor (EGF) to PE40, which is the natural toxin domain of pseudomonas exotoxin A, or to PE24mut, its de-immunized variant. In EGFR-expressing prostate carcinoma cells, both conjugates inhibited protein synthesis and induced apoptosis in a concentration- and time-dependent manner, with IC_50_ values in the nanomolar/picomolar range. Interestingly, both conjugates were 600–140,000-fold more cytotoxic than the EGFR inhibitor erlotinib [13].

In conclusion, the studies collected in this Special Issue confirm the efficacy and the potentiality of toxins as payloads of immunotoxins/conjugates designed for targeted therapy on several cancer models. Moreover, the knowledge of the structural and binding characteristics of toxins and related conjugates, together with the elucidation of their mechanism(s) of action, provide useful information for better pharmacological strategies with the aim of achieving a higher specificity and potency in targeting and destroying cancer cells.
